# Changes in blood pressure and related risk factors among nurses working in a negative pressure isolation ward

**DOI:** 10.3389/fpubh.2022.942904

**Published:** 2022-07-22

**Authors:** Yaoyao Wang, Junzhang Tian, Hongying Qu, Lingna Yu, Xiaoqin Zhang, Lishan Huang, Jianqun Zhou, Wanmin Lian, Ruoting Wang, Lijun Wang, Guowei Li, Li Tang

**Affiliations:** ^1^Department of Public Health and Preventive Medicine, School of Medicine, Jinan University, Guangzhou, China; ^2^Institute for Healthcare Artificial Intelligence Application, Guangdong Second Provincial General Hospital, Guangzhou, China; ^3^Center for Clinical Epidemiology and Methodology (CCEM), Guangdong Second Provincial General Hospital, Guangzhou, China; ^4^Nursing Department, Guangdong Second Provincial General Hospital, Guangzhou, China; ^5^Infectious Diseases Ward, Guangdong Second Provincial General Hospital, Guangzhou, China; ^6^Center for Information, Guangdong Second Provincial General Hospital, Guangzhou, China; ^7^Department of Health Research Methods, Evidence, and Impact (HEI), McMaster University, Hamilton, ON, Canada

**Keywords:** COVID-19, negative pressure isolation ward, blood pressure, nurse, risk factors

## Abstract

**Objective:**

To observe changes in blood pressure (ΔBP) and explore potential risk factors for high ΔBP among nurses working in a negative pressure isolation ward (NPIW).

**Methods:**

Data from the single-center prospective observational study were used. Based on a routine practice plan, female nurses working in NPIW were scheduled to work for 4 days/week in different shifts, with each day working continuously for either 5 or 6 h. BP was measured when they entered and left NPIW. Multivariable logistic regression was used to assess potential risk factors in relation to ΔBP ≥ 5 mm Hg.

**Results:**

A total of 84 nurses were included in the analysis. The ΔBP was found to fluctuate on different working days; no significant difference in ΔBP was observed between the schedules of 5 and 6 h/day. The standardized score from the self-rating anxiety scale (SAS) was significantly associated with an increased risk of ΔBP ≥ 5 mm Hg (odds ratio [OR] = 1.12, 95% CI: 1.00–1.24). Working 6 h/day (vs. 5 h/day) in NPIW was non-significantly related to decreased risk of ΔBP (OR = 0.70), while ≥ 2 consecutive working days (vs. 1 working day) was non-significantly associated with increased risk of ΔBP (OR = 1.50).

**Conclusion:**

This study revealed no significant trend for ΔBP by working days or working time. Anxiety was found to be significantly associated with increased ΔBP, while no <2 consecutive working days were non-significantly related to ΔBP. These findings may provide some preliminary evidence for BP control in nurses who are working in NPIW for Coronavirus Disease 2019 (COVID-19).

## Introduction

Due to the high risk of virus transmission for Coronavirus Disease 2019 (COVID-19), the negative pressure isolation ward (NPIW) becomes one of the main battlefields to treat patients with COVID-19 and control nosocomial infection (1). Nurses are on the front lines of caring for patients with COVID-19 and delivering the primary interventions that many patients receive while waiting for individualized treatment and recovery (2). Nurses have to wear heavy protective clothing to enter NPIW to minimize the risk of nosocomial infection, thereby seriously disrupting their normal work and life (3). With the full set of the protective equipment, their breathing and mobility are limited; for instance, they do not drink water or use the washroom to avoid the waste of time and some disposable equipment (4). The demanding work in NPIW therefore could easily increase nurses' psychological and physical stress, especially given their workload, long-term fatigue, and fear of getting infected (5).

Several studies have investigated mental health, such as depression, anxiety, and stress, of nurses treating patients with COVID-19 (6–8). Some studies had advocated more efforts and resources should be targeted on nurses' health and nursing trials (9, 10). While previous research consistently showed that high occupational and psychosocial stress among nurses may contribute to the development and exacerbation of hypertension (11, 12); there was sparse and limited evidence on nurses' physical health, especially for those working in NPIW. Enhancing nurses' physical health can help to improve their work quality in NPIW, ensure adequate patient care, and minimize the risk of nosocomial infection (13, 14).

Therefore, in this study, we evaluated the changes in blood pressure (ΔBP) for the nurses working in NPIW and explored potential factors related to their ΔBP. Results from this study were expected to provide preliminary evidence for improving nurses' physical health while working in NPIW for the COVID-19 combat.

## Methods

### Study setting and participants

This was a single-center prospective observational study conducted between 20 February and 17 May 2020 in Guangdong Second Provincial General Hospital located in Guangzhou, China. The target population was nurses working in the NPIW. Convenience sampling was used in this study for nurse enrollment. We included nurses who were working in NPIW against COVID-19 and agreed to participate. Those with pregnancy were excluded. This study was approved by the Institutional Review Board of Guangdong Second Provincial General Hospital. Written informed consent was obtained from all participating nurses.

### Study procedure

**Figure 1:**
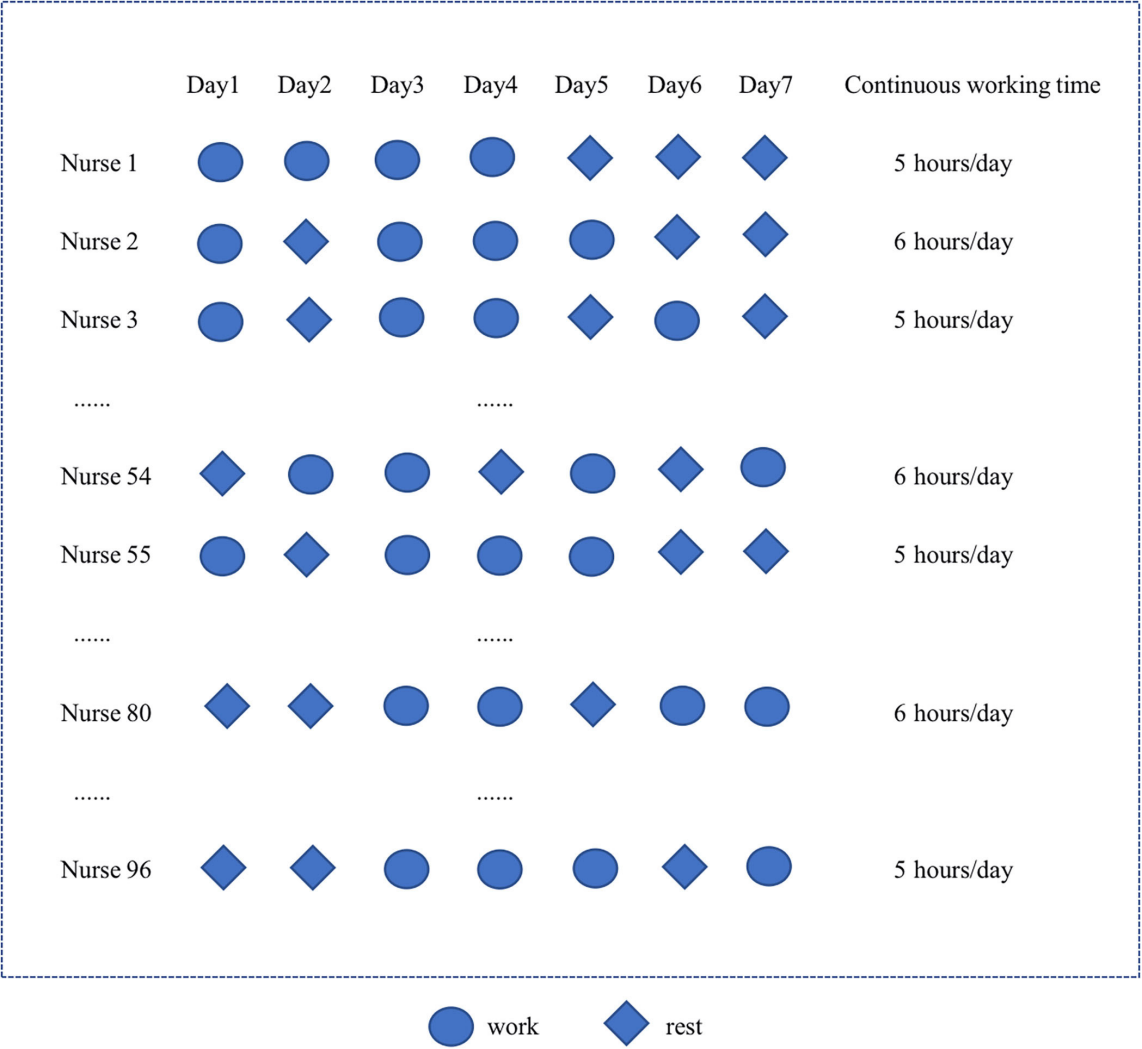
Illustration of the working schedule for the participating nurses in negative pressure isolation ward (NPIW).

Based on a routine practice plan, nurses were scheduled to work for 4 days per week (working Days 1–4) for the different shifts (day, afternoon, evening, and night shift) in the NPIW, with each day working continuously for either 5 or 6 h. While their work shifts were rotated on different working days, the working time (5 or 6 h/day) was fixed through the weekly schedule. Figure 1 depicts the illustration of the working schedule on a weekly basis for the participating nurses in NPIW.

According to the recommendation that the number of events was at least 10 times the number of exploratory variables in a fitted logistic regression model (15), we expected 5 exploratory factors would be included in the model. Therefore, a number of 50 nurses with events (ΔBP ≥ 5 mm Hg, defined below) would be required. Based on our previous experience, we conservatively estimated that the proportion of nurses whose ΔBP ≥ 5 mm Hg was no <60%. Given that some samples may be unavailable for analysis, an extra 10% was taken into consideration in the sample size estimation. Thus, a minimum sample size of 92 nurses was expected for enrollment.

Before entering the NPIW, data on self-administered demographic questionnaires, the self-rating anxiety scale (SAS), sleep time and quality, and the self-rating depression scale (SDS) were documented for each participating nurse. Data on nurses' sleep quality were collected by asking them “In general, what do you think about your sleep quality?” with a response option of either *high* or *low*. The self-rating anxiety and depression scales were both 20-item self-rating tools, in which each item received a score of 1, 2, 3, or 4 points to reflect the severity of syndromes (16, 17). Subsequently, the scores for each of the 20 items were added together for a total crude score that ranged from 20 to 80 points. A standardized score was obtained by taking the integer after multiplying the crude score by 1.25, ranged from 25 to 100 points, with a higher score representing a greater degree of depression or anxiety.

Systolic blood pressure (SBP) and diastolic blood pressure (DBP) were measured when they entered and left NPIW. Nurses' BP was measured with an electronic sphygmomanometer. Equipment was calibrated by the same trained researcher upon nurses' entry into the NPIW. All nurses' BP was measured by the same electronic sphygmomanometer and their results were checked by the same investigator throughout the study.

### Outcome measure

Our outcome was the ΔBP ≥ 5 mmHg when participating nurses were working in NPIW, in which the selection of 5 mmHg was based on the literature in combination with our clinical expertise (18, 19). To enhance simplicity and straightforwardness, we categorized nurses who were in a “high change in SBP [ΔSBP]” group or “high change in DBP [ΔDBP]” group into the “high ΔBP” group and the others into the control group.

To determine high ΔSBP and ΔDBP groups, first, we calculated the ΔSBP and ΔDBP for each working day by using the BP values when nurses left NPIW minus the BP values when they entered NPIW. Then the maximum values of ΔSBP and ΔDBP for the four working days were selected. Subsequently, the maximum ΔSBP and ΔDBP of ≥ 5 mmHg were assigned to a “high ΔSBP” group and “high ΔDBP” group, respectively, while the others were assigned to a control group.

### Independent variables

Details on the independent variables for nurses working in NPIW were described as follows: age (in years), standardized SDS score, standardized SAS score, sleep time (in hours), body mass index (BMI; categorized as normal weight, underweight, and overweight), married (never married, married, or other), had any child (yes and no), self-reported health (bad, moderate, and good), ever worked in NPIW (yes and no), consecutive working days (<2 days and no <2 days), working hours scheduled in NPIW (5 and 6 h), sleep quality classification (high and low), and menstruation (yes and no).

### Statistical analysis

We described continuous variables with mean and standard deviation (SD) and categorical variables with counts and percentages. We used the analysis of covariance (ANCOVA) to assess whether there was a significant difference in ΔBP between the 4 working days, where the daily ΔBP was from the higher value between ΔSBP and ΔDBP. The Student's *t*-test was employed to compare whether the ΔBP significantly differed between nurses scheduled for working 5 and 6 h/day.

Univariate logistic regression analyses were first used to assess the relationship between ΔBP ≥ 5 mm Hg and the aforementioned independent variables. Based on clinical experience and group discussion, we also selected a total of 5 exploratory factors (age, working hours scheduled in NPIW, consecutive working days, ever worked in NPIW, and standardized SAS score) into the multivariable logistic model. We performed a sensitivity analysis by using the ΔBP ≥ 5 mmHg for each working day as the outcome, i.e., the nurses were first categorized into the high ΔBP group if they had a ΔSBP or ΔDBP value of ≥5 mmHg on each of the 4 working days; subsequently we used a generalized estimated equation (GEE) model with a logit function after adjusting for age, working hours scheduled in NPIW, consecutive working days, ever worked in NPIW, and standardized SAS score. All results were shown as odds ratios (ORs) and their corresponding 95% confidence intervals (95% CIs).

A two-sided *p* < 0.05 was considered statistically significant. All analyses were performed using Stata/SE 15.1.

## Results

We enrolled 96 nurses in this study, among whom 12 had no data on BP available. Therefore, a total of 84 nurses were included in the analyses. Their baseline characteristics are shown in Table 1. The mean age was 29.0 years (SD: 5.65) and the mean sleep time was 7.2 h (SD: 0.92). The average standardized scores from SDS and SAS were 42.6 (SD: 9.51) and 40.9 (SD: 7.51), respectively. In total, 40.5% of the nurses had never worked in NPIW before. None of the nurses had a previous diagnosis of hypertension. There were 62.5% of the nurses who worked for 5 h/day in NPIW and 37.5% for 6 h/day. Less than a half (47.5%, *n* = 38) were scheduled to work no <2 consecutive days in NPIW: 10 (26.3%), 15 (39.5%), and 13 (34.2%) worked 2, 3, and 4 consecutive days, respectively.

**Table 1:** Description of baseline characteristics for the 84 included nurses working in NPIW.

**Variables**	**Description**
Age: Mean (SD), in years	29.01 (5.65)
Standardized SDS score: Mean (SD)	42.56 (9.51)
Standardized SAS score: Mean (SD)	40.86 (7.51)
Sleep time: Mean (SD), in hours	7.21 (0.92)
BMI classification: *n* (%), in kg/m^2^	
Normal weight	56 (66.67)
Underweight	23 (27.38)
Overweight	5 (5.95)
Married: *n* (%)	
Never married	46 (54.76)
Married or other	38 (45.24)
Had any child: *n* (%)	
No	53 (63.10)
Yes	31 (36.90)
Self-reported health: *n* (%)	
Bad	3 (3.57)
Moderate	62 (73.81)
Good	19 (22.62)
Ever worked in NPIW: *n* (%)	
Yes	50 (59.52)
No	34 (40.48)
Consecutive working days: *n* (%)	
<2 days	42 (52.50)
No <2 days	38 (47.50)
Working hours scheduled in NPIW: *n* (%)	
5 h	50 (62.50)
6 h	30 (37.50)
Sleep quality classification: n (%)	
High	53(63.09)
Low	31(36.90)
Menstruation: *n* (%)	
Yes	34 (40.96)
No	49 (59.04)

*SDS, self-rating depression scale; BMI, body mass index; SAS, self-rating anxiety scale; SD, standard deviation; NPIW, negative pressure isolation ward*.

### ΔBP for nurses in NPIW

**Figure 2:**
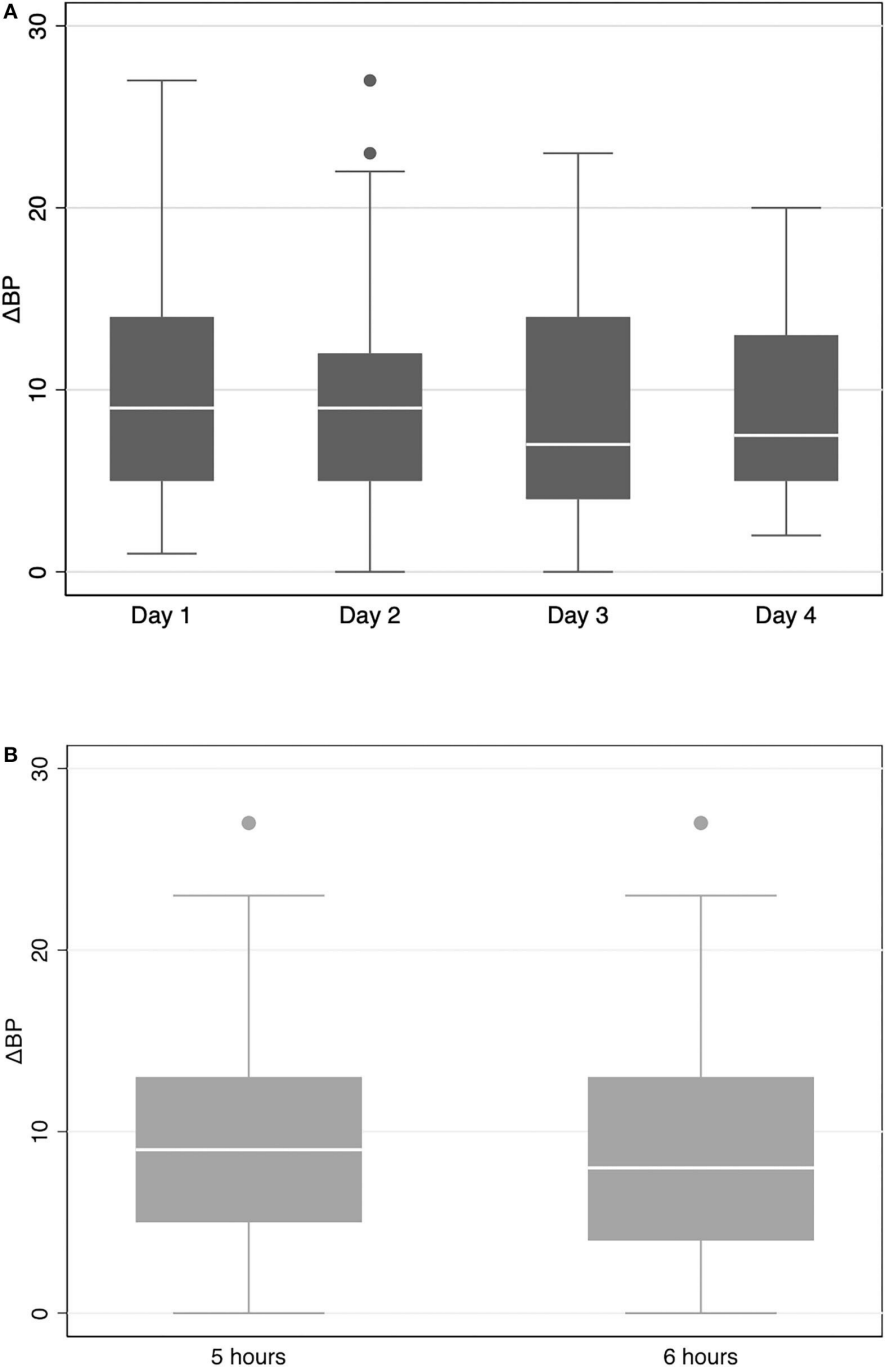
Box plot of the variation of changes in blood pressure (ΔBP) among nurses in negative pressure isolation ward (NPIW) [**(A)** stratified by working day; **(B)** stratified by working hours].

As displayed in Figure 2A, the ΔBP is found to fluctuate on different working days, ranging from 7 mmHg (on Day 3) to 9 mmHg (on Day 2), with a *p* of 0.42 from the ANCOVA. No significant difference in ΔBP was observed between the schedule of 5 and 6 h/day (9 vs. 8 mmHg, *p* = 0.74; Figure 2B). Similar trends in working days and working time scheduled were also found for ΔSBP and ΔDBP (Supplemental Figures S1–S3).

### Relationship between exploratory variables and high ΔBP

There were 64 nurses (76.2%) with a ΔBP ≥ 5 mm Hg during the study; therefore, they were categorized into the high ΔBP group. Table 2 shows the results from univariate logistic regression analysis for the relationship between the exploratory variable and ΔBP. The standardized SAS score and low sleep quality were associated with an increased risk of high ΔBP, with an OR of 1.12 (95% CI: 1.01–1.23) and 5.44 (95% CI: 1.14–25.95), respectively. Age and working no <1 consecutive day were non-significantly associated with an increased risk of high ΔBP. By contrast, 6 h/day in NPIW and ever worked in NPIW before were non-significantly related to a decreased risk of ΔBP. Supplemental Table S1 displays that there is no statistically significant relationship between variables and ΔSBP. Overweight was related to a decreased risk of high ΔDBP when compared with normal weight (OR = 0.34, 95% CI: 0.12–0.99), while low sleep quality was associated with an increased risk (OR = 3.93, 95% CI: 1.29–11.93; Supplemental Table S2).

**Table 2:** Univariate analysis for the relationship between variables and ΔBP for nurses working in NPIW.

**Variables**	**OR**	**95%CI**	***P***
Working hours scheduled in NPIW^a^	0.72	0.24–2.19	0.565
No <2 consecutive working days^b^	1.21	0.40–3.64	0.737
Age (years)	1.02	0.92–1.13	0.708
Married^c^	0.94	0.31–2.83	0.910
Had any child^d^	0.67	0.22–2.05	0.487
BMI			
Underweight	1.42	0.35–5.71	0.624
Overweight	0.38	0.06–2.53	0.313
Standardized SDS score	1.01	0.95–1.08	0.672
Standardized SAS score	1.12	1.01–1.23	0.029
Inadequate sleep time^e^	1.09	0.31–3.83	0.899
Ever worked in NPIW^f^	0.38	0.11–1.30	0.122
Low sleep quality^g^	5.44	1.14–25.95	0.033
Menstruation^h^	0.94	0.31–2.83	0.910
Work shift			
Afternoon	0.86	0.25–2.90	0.802
Evening	0.58	0.09–3.65	0.565
Night	1.63	0.17–15.51	0.669

*ΔBP, change in blood pressure; SDS, self-rating depression scale; BMI, body mass index; SAS, self-rating anxiety scale; SD, standard deviation; NPIW, negative pressure isolation ward*.

*^a^Taking 5 h as a reference; ^b^taking 1 consecutive day as a reference; ^c^taking never married as a reference; ^d^taking no child as a reference; ^e^taking sleep time in 7–9 h as a reference; ^f^taking never worked in NPIW as a reference; ^g^taking high as a reference; and ^h^taking no as a reference*.

Results from the multivariable logistic regression found that a standardized SAS score was significantly associated with a 12% increased risk of high ΔBP (OR = 1.12, 95% CI: 1.00–1.24; Table 3). Working 6 h/day (vs. 5 h/day) in NPIW was non-significantly related with decreased risk of high ΔBP (OR = 0.70, 95% CI: 0.21–2.38), while ≥ 2 consecutive working days (vs. 1 working day) was non-significantly associated with high ΔBP (OR = 1.50, 95% CI: 0.44–5.11). Likewise, age and ever worked in NPIW were not significantly related to the risk of ΔBP. As shown in Supplemental Table S3, no significant association between the exploratory factors and ΔSBP and ΔDBP is observed from the multivariable logistic regression analyses.

**Table 3:** Multivariable analysis for the relationship between exploratory factors and ΔBP for nurses working in NPIW.

**Exploratory factors**	Δ**BP**	
	**OR (95% CI)**	***P***
Age (years)	1.03 (0.93–1.15)	0.532
Working hours scheduled in NPIW		
5 h	Reference	
6 h	0.70 (0.21–2.38)	0.571
Consecutive working days		
<2 days	Reference	
≥2 days	1.50 (0.44–5.11)	0.521
Ever worked in NPIW		
No	Reference	
Yes	0.44 (0.12–1.59)	0.210
Standardized SAS score	1.12 (1.00–1.24)	0.045

*ΔBP, change in blood pressure; OR, odds ratio; NPIW, negative pressure isolation ward; SAS, self-rating anxiety scale*.

Table 4 demonstrates similar results from sensitivity analysis by using the GEE for the main findings. Working 6 h/day and ever worked in NPIW were non-significantly related to decreased risk of high ΔBP, while standardized SAS score and ≥ 2 consecutive working days were non-significantly associated with the increased risk of ΔBP. Similar results from sensitivity analyses were also observed for ΔSBP and ΔDBP to the main analyses (Supplemental Table S4).

**Table 4:** Sensitivity analysis by the generalized estimated equation for the relationship between exploratory factors and ΔBP for nurses working in NPIW.

**Exploratory factors**	Δ**BP**	
	**OR (95% CI)**	***P***
Age (years)	1.01 (0.96–1.05)	0.697
Working hours scheduled in NPIW		
5 h	Reference	
6 h	0.79 (0.45–1.39)	0.416
Consecutive working days		
<2 days	Reference	
≥2 days	1.23 (0.74–2.04)	0.433
Ever worked in NPIW		
No	Reference	
Yes	0.62 (0.37–1.04)	0.069
Standardized SAS score	1.03 (0.99–1.07)	0.089

*ΔBP, change in blood pressure; OR, odds ratio; NPIW, negative pressure isolation ward; SAS, self-rating anxiety scale*.

## Discussion

In this prospective observational study for nurses working in NPIW, we found that their ΔBP fluctuated on different working days and working times. The standardized SAS score was significantly associated with an increased risk of high ΔBP, while 6 h/day and ≥ 2 consecutive working days were not significantly related to high ΔBP.

We found no significant trend in ΔBP through different working days and working times. Several potential interpretations may exist. The nurses were relatively young and healthy (Table 1), therefore, their ΔBP variability may be small or could even regress to the mean after they got used to and adapted to the work in NPIW. The small sample with a short study time may also fail to observe a true pattern of the ΔBP. Nevertheless, our results required further high-quality research for validation and clarification of the trend in ΔBP for nurses working in NPIW.

Since the outbreak of COVID-19, nurses had fought against the virus and taken care of the infected patients. Consequently, they suffered from the risk of infection and acute psychological effects, such as depression, anxiety, and post-traumatic stress disorder. Some previous studies exploring a series of psychological problems for healthcare workers during the pandemic found that the incidence of anxiety and depression had the highest rates (20, 21). One study showed that anxiety was strongly associated with BP increment (22). However, the protective equipment and regulations in NPIW inevitably increased nurses' anxiety, thereby impairing their physical health. Therefore, our study findings emphasized the continuous monitoring and long-term intervention of nurses' psychological problems, which was essential to enhance their physical health outcomes (23).

More than 40% of the nurses in this study had never worked in NPIW before, which may commonly occur worldwide, given the rapid spread of severe acute respiratory syndrome coronavirus 2 (SARS-CoV-2). Therefore, they tended to have a high risk of increased BP in NPIW due to lack of training, fatigue, burnout, and fear of nosocomial infection (24). Likewise, the rocketing number of patients posed an overwhelming demand to the healthcare system, requiring nurses to continuously work without an adequate break (25). Our results revealed that working consecutively for no <2 days in NPIW was associated with high ΔBP, which supported the avocation of scientific and evidence-based arrangement for the nurses on the front lines. Moreover, unduly long-time and high-load work could significantly affect the overall work quality for patients, healthcare workers, and the system (26). However, when compared to 5 h/day, we found that 6 h/day was related to decreased risk of high ΔBP, although the relationship was not significant. As mentioned above, similar interpretations that included the young and robust nurses enrolled, the sample size, and the short observational time may result in a large variance in the model to impact the association. However, how to reasonably schedule working time in NPIW and whether working for 6 h/day would reduce the ΔBP, remained further clarification and exploration.

Although most research for nurses during COVID-19 focused on mental health, no previous study reported findings on BP for those working in NPIW. For instance, one study investigated nurses' anxiety and the related factors during the early stage of COVID-19 in Wuhan (27). Another study in Turkey explored the burnout and sleep quality of nurses caring for patients with COVID-19 (28). Their results were consistent with that the pandemic had imposed a tremendous impact on nurses' psychological function (11, 29, 30), yet without evidence of their physical health provided. To our best knowledge, this is the first study on BP among nurses working in NPIW. Unlike the psychological information mainly obtained from questionnaires and scales, ΔBP acted as an objective measure for physical reaction and health indicator. For example, ΔBP had been used as a surrogate associated with the risk of multiple diseases that included cardiovascular diseases, kidney dysfunction, and even mortality (31, 32). Some studies also used the qualitative methods with interviews to explore the working environment and shift patterns in relation to nurses' experience and perception of working in NPIW (33, 34). While their outcomes tended to be subjective, no data on nurses' physical health could be generated. Therefore, our study may provide some preliminary evidence for BP control in nurses who were working in NPIW for COVID-19.

Our study findings may have implications for scientific policy-making regarding alleviating the heavy physical burden on nurses who were working in NPIW. The sound methodology and analyses supported the accuracy of our results. Some limitations need to be acknowledged. First, the assessment of reliability and validity of measurement tools was critically important to the study findings; unfortunately, no formal evaluation of the reliability and validity was conducted in this study. Second, random sampling was not feasible and applicable in NPIW during the pandemic. Therefore, the use of convenience sampling may compromise the generalizability of our findings and impair the validity to some unknown extent. Likewise, the included nurses were exclusively women, thereby limiting the generalizability of findings to male nurses. The relatively small sample size precluded us from further exploration and subgroup analyses. Moreover, as an observational study, residual confounding would be inevitable, and no causal relationship could be identified.

## Conclusion

In this study, we found no significant trend for ΔBP by working days and working time. Anxiety was found to be significantly associated with increased ΔBP, while no <2 consecutive working days were non-significantly related to ΔBP. These findings may provide some preliminary evidence for policy-making regarding BP control in nurses who were working in NPIW for COVID-19.

## Data availability statement

The raw data supporting the conclusions of this article will be made available by the authors, without undue reservation.

## Ethics statement

The studies involving human participants were reviewed and approved by the Institutional Review Board of Guangdong Second Provincial General Hospital (No. 2020-15-01-YXKXYJ-CRB). The patients/participants provided their written informed consent to participate in this study.

## Author contributions

YW, JT, HQ, LY, XZ, LT, and GL collected and analyzed the data. YW, JT, HQ, LY, and XZ drafted the manuscript. LT and GL made critical revisions to the draft. LH, JZ, WL, RW, and LW provided professional help with manuscript writing and revisions. All the authors designed the study, read, and approved the final manuscript.

## Funding

This study was funded by the Science Foundation of Guangdong Second Provincial General Hospital (Grant recipient: GL; Grant No.: YY2018–002), the Science and Technology Program of Guangzhou (Grant recipient: GL; Grant No.: 202002030252), R&D Plan Project of Key Fields in Guangdong Province (the 7th Batch) (Grant recipient: JT; Grant No.: 2020B0101130020), and the National Key R&D Program of China (Grant recipient: JT; Grant No.: 2021YFC2009400).

## Conflict of interest

The authors declare that the research was conducted in the absence of any commercial or financial relationships that could be construed as a potential conflict of interest.

## Publisher's note

All claims expressed in this article are solely those of the authors and do not necessarily represent those of their affiliated organizations, or those of the publisher, the editors and the reviewers. Any product that may be evaluated in this article, or claim that may be made by its manufacturer, is not guaranteed or endorsed by the publisher.

## Abbreviations

BP: blood pressure
ΔBP: change in blood pressure
NPIW: negative pressure isolation ward
COVID-19: Coronavirus Disease 2019
SAS: self-rating anxiety scale
SDS: self-rating depression scale
SBP: systolic blood pressure
DBP: diastolic blood pressure
BMI: body mass index
SD: standard deviation
ANCOVA: analysis of covariance
GEE: generalized estimated equation
OR: odds ratio
95% CI: 95% confidence interval.
