# Supplementary material for: Changes in blood pressure and related risk factors among nurses working in a negative pressure isolation ward
Source: Front Public Health. 2022 Jul 22;10:942904. doi: 10.3389/fpubh.2022.942904 (PMC9353044; doi:10.3389/fpubh.2022.942904)

Supplemental Table 1. Univariate analysis for the relationship between variables and ΔSBP for nurses working in NPIW

Variables	OR	95%CI	*P*
Working hours scheduled in NPIW[1]	0.89	0.35-2.29	0.809
No less than two consecutive working days[2]	1.07	0.43-2.68	0.888
Age(years)	1.04	0.96-1.13	0.359
Married[3]	0.89	0.35-2.26	0.802
Had any child[4]	1.32	0.50-3.48	0.575
BMI			
Underweight	0.98	0.33-2.87	0.971
Overweight	0.79	0.12-5.15	0.807
Standardized SDS score	1.03	0.98-1.09	0.201
Standardized SAS score	1.04	0.98-1.21	0.151
Inadequate sleep time[5]	1.11	0.39-3.17	0.852
Ever worked in NPIW[6]	0.40	0.15-1.07	0.068
Low sleep quality[7]	1.43	0.54-3.76	0.469
Menstruation[8]	0.98	0.39-2.48	0.962
Work shift			
Afternoon	0.74	0.27-2.07	0.568
Evening	0.64	0.12-3.32	0.595
Night	1.44	0.25-8.22	0.682

Abbreviation: ΔSBP, change in blood pressure; SDS, Self-rating Depression Scale; BMI, Body Mass Index; SAS, Self-rating Anxiety Scale; SD, Standard Deviation; NPIW, Negative Pressure Isolation Ward.

[1] Taking 5 hours as reference; [2] Taking one consecutive day as reference; [3] Taking never married as reference; [4] Taking no child as reference; [5] Taking sleep time in 7 to 9 hours as reference; [6] Taking never worked in NPIW as reference; [7] Taking high as reference, low vs high;[8] Taking no menstruation as reference.

Supplemental Table 2. Univariate analysis for the relationship between variables and ΔDBP for nurses working in NPIW

Variables	OR	95%CI	*P*
Working hours scheduled in NPIW[1]	0.40	0.15-1.04	0.061
No less than two consecutive working days[2]	1.12	0.44-2.83	0.817
Age(years)	1.01	0.93-1.10	0.760
Married[3]	0.89	0.35-2.26	0.802
Had any child[4]	0.95	0.36-2.49	0.917
BMI			
Underweight	0.34	0.12-0.99	0.048
Overweight	0.23	0.03-1.51	0.125
Standardized SDS score	1.02	0.97-1.07	0.433
Standardized SAS score	1.06	0.99-1.15	0.063
Inadequate sleep time[5]	0.77	0.27-2.18	0.624
Ever worked in NPIW[6]	0.90	0.35-2.29	0.820
Low sleep quality[7]	3.93	1.29-11.93	0.016
Menstruation[8]	0.89	0.35-2.26	0.802
Work shift			
Afternoon shift	1.16	0.41-3.32	0.783
Evening shift	0.73	0.18-3.32	0.740
Night shift	1.04	0.17-6.54	0.964

Abbreviation: ΔDBP, change in blood pressure; SDS, Self-rating Depression Scale; BMI, Body Mass Index; SAS, Self-rating Anxiety Scale; SD, Standard Deviation; NPIW, Negative Pressure Isolation Ward.

[1] Taking 5 hours as reference; [2] Taking one consecutive day as reference; [3] Taking never married as reference; [4] Taking no child as reference; [5] Taking sleep time in 7 to 9 hours as reference; [6] Taking never worked in NPIW as reference; [7] Taking high as reference;[8] Taking no menstruation as reference.

Supplemental Table 3. Multivariable analysis for the relationship between exploratory factors and ΔSBP and ΔDBP for nurses working in NPIW

Exploratory factors	ΔSBP		ΔDBP	
	OR (95% CI)	*P*	OR (95% CI)	*P*
Age (years)	1.05 (0.96-1.15)	0.244	1.02 (0.93-1.11)	0.679
Working hours scheduled in NPIW				
5 hours	reference		reference	
6 hours	0.87 (0.31-2.42)	0.789	0.40 (0.15-1.10)	0.076
Consecutive working days				
< 2 days	reference		reference	
≧2 days	1.11 (0.42-2.96)	0.832	1.23 (0.46-3.32)	0.679
Ever worked in NPIW				
No	reference		reference	
Yes	0.96 (0.35-1.15)	0.091	0.96 (0.35-2.63)	0.930
Standardized SAS score	1.05 (0.98-1.12)	0.182	1.07 (0.99-1.15)	0.069

Abbreviation: ΔSBP, change in Systolic Blood Pressure; ΔDBP, change in Diastolic Blood Pressure; OR, Odds Ratio; NPIW, Negative Pressure Isolation Ward; SAS, Self-rating Anxiety Scale.

Supplemental Table 4. Sensitivity analysis by generalized estimate equation for the relationship between exploratory factors and ΔSBP and ΔDBP for nurses working in NPIW

Exploratory factors	ΔSBP		ΔDBP	
	OR (95% CI)	*P*	OR (95% CI)	*P*
Age (years)	1.03 (0.98-1.08)	0.280	0.98 (0.94-1.03)	0.439
Working hours scheduled in NPIW				
5 hours	reference		reference	
6 hours	0.72 (0.40-1.30)	0.281	0.75 (0.44-1.27)	0.285
Consecutive working days				
< 2 days	reference		reference	
≧ 2 days	1.11 (0. 64-1.93)	0.714	1.06 (0.67-1.69)	0.802
Ever worked in NPIW				
No	reference		reference	
Yes	0.51 (0.29-0.89)	0.018	0.73 (0.46-1.17)	0.196
Standardized SAS score	1.01 (0.98-1.05)	0.433	1.03 (1.00-1.06)	0.024

Abbreviation: ΔSBP, change in Systolic Blood Pressure; ΔDBP, change in Diastolic Blood Pressure; OR, Odds Ratio; NPIW, Negative Pressure Isolation Ward; SAS, Self-rating Anxiety Scale.

Supplemental Figure 1. Box plot of variation of ΔSBP among nurses stratified by working day in NPIW

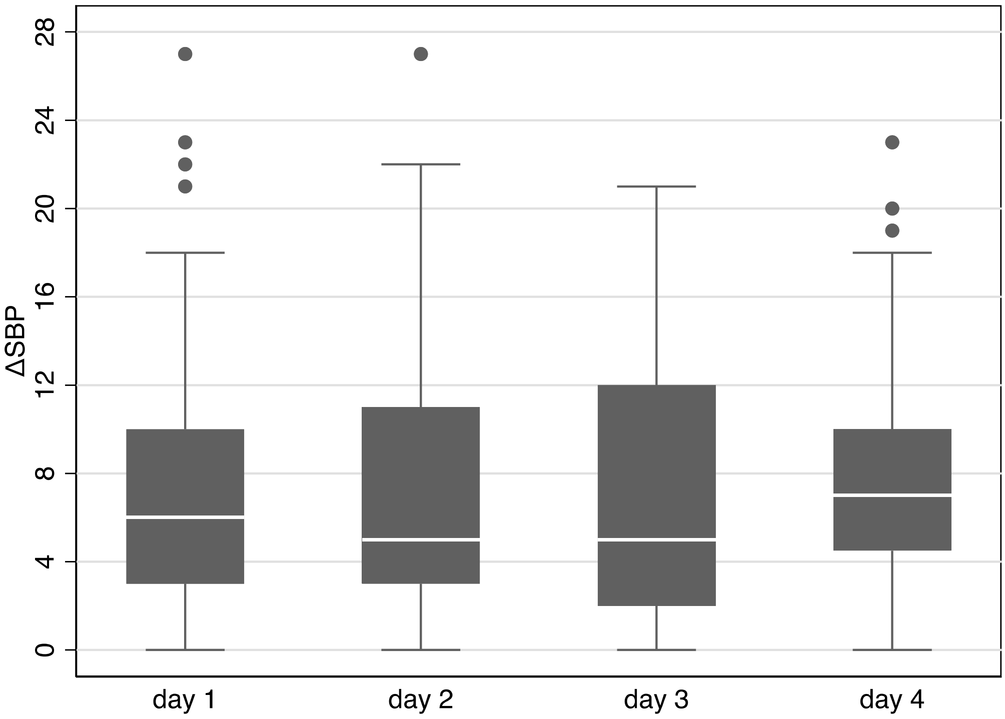

Supplemental Figure 2. Box plot of variation of ΔDBP among nurses stratified by working day in NPIW

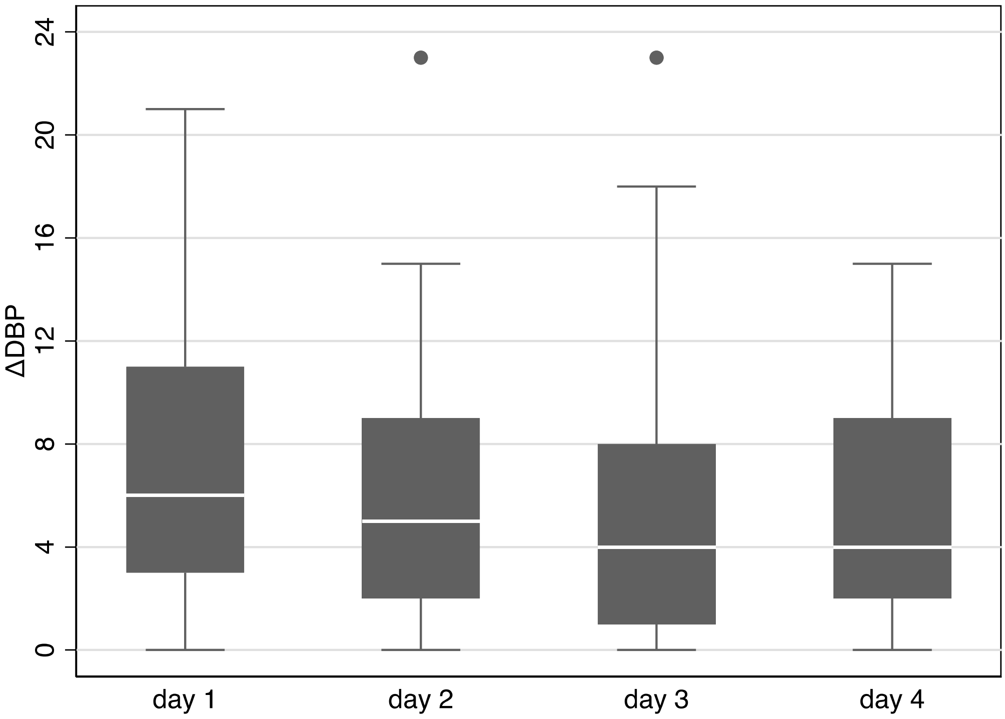

Supplemental Figure 3. Box plot of variation of ΔSBP and ΔDBP among nurses stratified by working hours scheduled in NPIW

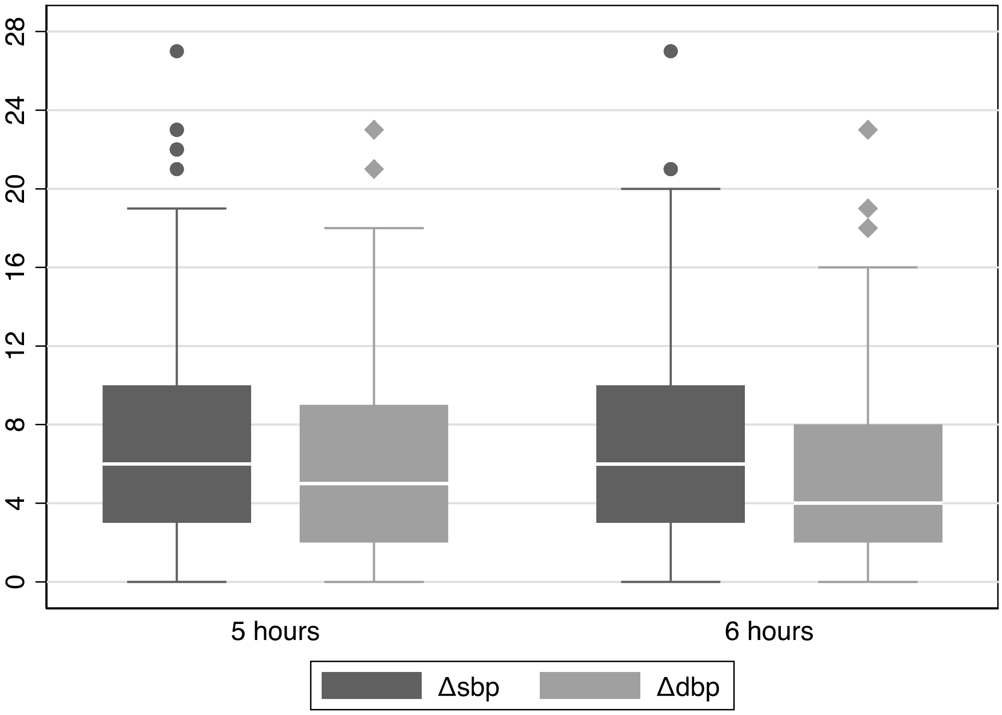

Supplement: Supplementary file 1 [file Data_Sheet_1.pdf]
